# Educational–representational alignment among MD/MBA graduates in US Senior Academic Medical Leadership: a cross-sectional study

**DOI:** 10.1186/s12909-026-09039-4

**Published:** 2026-04-06

**Authors:** Christopher J. Shin, Tarik F. Massoud

**Affiliations:** 1 Program in Science, Technology and Society, Stanford University School of Humanities and Sciences, 450 Jane Stanford Way, Building 200, Room 19, Stanford, CA 94305 USA; 2 Division of Neuroimaging and Neurointervention, Department of Radiology, Stanford University School of Medicine, Stanford Center for Academic Medicine, Radiology MC: 5659453 Quarry Road, Palo Alto, CA 94304 USA

**Keywords:** MD/MBA programs, Academic medical leadership, Dual-degree education, Leadership-role alignment, Professional identity formation

## Abstract

**Background:**

MD/MBA programs have expanded rapidly in the United States, providing formal training in management and organizational principles relevant to clinical and institutional practice. However, it remains unclear how this dual-degree training is reflected in senior academic medical leadership positions. This study evaluates how MD/MBA graduates are deployed as senior leaders and compares them with MBA-only administrators and MD-only physician leaders. The authors hypothesize that MD/MBA holders would be more frequently represented in finance, operations, and other system-facing roles relative to MD-only leaders.

**Methods:**

This study provided a cross-sectional analysis of senior leaders at the 50 U.S. medical schools receiving the most NIH funding in 2024. Public organizational charts and biographies identified educational backgrounds, leadership roles, and demographic information for deans, vice deans, associate deans, and assistant deans. Leadership titles were coded into 16 categories and then collapsed into six domains. Three groups were selected for comparative analysis: MD/MBA, MBA-only, and MD-only individuals. Descriptive statistics and a 3 × 6 Pearson chi-square test assessed differences in leadership placement; pairwise Fisher’s exact tests compared likelihoods of holding system-facing roles; and demographic comparisons used chi-square tests. *p* < 0.05 defined significance.

**Results:**

Of 1,300 individuals screened, 634 met inclusion criteria (22 MD/MBA, 38 MBA-only, 574 MD-only). Leadership placement differed significantly across groups (χ²(10) = 119.5, *p* < 0.0001). MBA-only leaders were concentrated in system-facing roles (25/38), whereas MD/MBA graduates rarely held such positions (3/22) and resembled MD-only leaders (48/574) who were primarily assigned to educational and clinically-centered domains. MBA-only leaders were significantly more likely than MD/MBA (OR 12.18, 95% CI 3.03–48.9; *p* = 0.0004) or MD-only leaders (OR 21.07, 95% CI 10.12–43.85; *p* < 0.0001) to hold system-facing roles (i.e., school-level executive leadership roles involving finance, operations, or institutional administration). No demographic differences were found.

**Conclusions:**

MD/MBA graduates were not more frequently represented in systems-facing roles than MD-only peers, suggesting that other factors, including professional identity and organizational norms, may outweigh degree attainment in leadership role placement decisions. These findings highlight a possible need for clearer integration of management preparation into physician leadership pathways.

## Background

MD/MBA dual-degree programs have grown substantially over the past two decades, combining medical education with formal graduate-level training in business disciplines relevant to clinical and organizational settings. The number of US medical schools offering MD/MBA programs has risen from 33 in 2002 to 92 in 2022 [1], which indicates sustained interest in educational models that integrate medical training with managerial and systems-level (i.e., institutional operations, finance, and executive administration) competencies. From an educational standpoint, MD/MBA programs function as structured pathways that integrate medical training with formal instruction in organizational and business principles. They provide an opportunity to examine how degree attainment is associated with leadership role placement in academic medicine.

Prior research describes who pursues MD/MBA training and why. MD/MBA students commonly identify gaps in traditional medical training, viewing the MBA curriculum instead as offering organizational, financial, and leadership competencies not addressed in medical school [2]. Larson et al. document how increasing administrative and financial complexity in US health care creates demand for physicians with formal management preparation, which motivates the rise of MD/MBA programs [3]. Together, these studies establish the educational rationale for dual-degree pathways. These programs provide physicians with training across both clinical and organizational domains.

However, little is known about how MD/MBA graduates are represented in senior leadership roles within academic medicine. Azzam et al. offer one of the few empirical windows into this question, showing that within the University of California medical system, 34.6% of physician leaders hold at least one dual degree compared to 25.3% of non-leaders, though MD/MBAs make up only 5.8% of these dual-degree leaders [4]. These findings suggest that while advanced degrees are valued, the MD/MBA remains a relatively uncommon credential among senior medical school leaders within the institutions studied. Although physicians with MBAs feel better equipped to navigate complex institutional demands concerning financial and administrative decision-making [5], it remains unclear whether individuals with MD/MBA degrees are more frequently promoted into system-facing leadership roles. Ackerly et al. observe that many physician leaders arrive at their positions “by accident” rather than through structured preparation [6], which underscores the absence of defined educational pathways into leadership within academic medicine.

Conceptual frameworks from medical education help to explain why training and leadership placement do not always align. Professional identity formation shapes how physicians come to understand the types of responsibilities that feel consistent with their clinical roles, which can make them gravitate toward teaching or student-facing work rather than institutional management [7]. The “hidden curriculum”, which refers to the informal lessons and expectations conveyed by the culture and routines of medical training, can further reinforce assumptions about who is expected to lead particular aspects of an organization [8]. Prior work on professional identity formation suggests that physicians internalize norms and values that emphasize clinical roles, and that transitions into administrative or organizational leadership roles may involve identity adjustment and negotiation of professional expectations [9]. These influences may shape how leadership roles are distributed among individuals with different degree backgrounds.

In parallel, many medical schools appoint non-clinical administrators with MBA training to leadership roles that involve institutional planning, financial oversight, and organizational strategy. These individuals represent an alternative educational pathway into system-oriented responsibilities and therefore serve as a natural comparison group. What remains unclear is how MD/MBA graduates are represented in leadership roles relative to MBA-only and MD-only leaders. Understanding how these groups are positioned relative to one another offers insight into how dual-degree training is interpreted and used within academic medicine.

To address this gap, the present study provides a cross-sectional analysis of senior leaders at the top 50 US medical schools receiving the most NIH funding, which has been used in prior analyses as a proxy for institutional research intensity and resource scale [10, 11]. Leadership roles occupied by MD/MBA leaders, MBA-only leaders, and MD-only leaders were compared. This study focuses specifically on senior leadership roles within schools of medicine at the 50 most NIH-funded US institutions and does not attempt to generalize beyond these settings. This study hypothesizes that MD/MBA graduates are more frequently represented in finance, operations, and other system-facing roles compared to MD-only peers and similarly represented compared to MBA-only administrators. By comparing MD/MBA graduates with MD- and MBA-only leaders, this study aims to assess how dual-degree credentials are associated with systems-oriented leadership roles within highly NIH-funded US medical schools and whether dual-degree training differentiates physicians in their likelihood of assuming system-oriented responsibilities.

## Methods

This study exclusively used publicly available institutional data and did not involve interaction with human participants or access to identifiable private information. In accordance with US federal regulations (45 CFR § 46.102), this work does not constitute human subjects research and was therefore exempt from institutional board review. All data collection and coding were conducted manually and reviewed for accuracy.

This study examined the 50 US medical schools that received the highest total NIH research funding in 2024, as reported by the Blue Ridge Institute for Medical Research (BRIMR) [10]. NIH funding rank provided an objective measure of institutional research intensity and size; prior analyses showed that the top 50 schools accounted for over 80% of all NIH funding awarded to US medical schools [11]. These highly NIH-funded medical schools manage large clinical, educational, and research enterprises that require substantive administrative coordination and clearly differentiated leadership roles [12]. The structural complexity of these institutions was considered an appropriate context in which to examine patterns of dean-level leadership representation. Focusing on these institutions also ensured that leadership structures were consistently documented and comparable across schools, which supported accurate classification of functional roles.

Leadership role placement was examined in relation to degree background. In this study, leadership placement was conceptualized as an observed institutional assignment rather than a measure of individual preparedness, professional competence, or the effectiveness of MD/MBA training. Leadership placement reflects institutional promotion and appointment decisions and may be influenced by individual career preferences, prior experiential qualifications, institutional culture, and organizational structures not directly measured in this analysis. Between June and August 2025, the official school of medicine website for each of the 50 institutions was accessed. Institutional navigation menus were used to identify pages titled “Leadership,” “Dean’s Office,” “Administration,” or equivalent. When necessary, institutional search functions were used with keywords such as “Dean,” “Vice Dean,” “Associate Dean,” and “Assistant Dean.” Leadership rosters and associated biological profiles published on each institution’s official school of medicine website were manually reviewed to record names, official titles, degree credentials, and described portfolio responsibilities. Each leader’s biography was individually examined to verify educational background and confirm primary functional emphasis. Only active dean-level appointments within the school of medicine were included. Individuals holding appointments exclusively with schools of dentistry, nursing, veterinary medicine, or other non-medical units were excluded, as were emeritus or honorary positions. Department chairs and hospital administrators without formal “dean” titles were not included in the analysis. The analysis was intentionally restricted to dean-level roles within schools of medicine to maintain structural comparability across institutions. Hospital and health system leadership structures vary substantially in reporting conventions, organizational integration with medical schools, and title standardization, which would introduce additional heterogeneity. Department chair roles were also excluded because departmental leadership responsibilities vary widely in scope and do not necessarily correspond to centralized, institution-wide administrative portfolios comparable to dean-level appointments. Focusing on dean-level positions allowed for consistent cross-institutional role classification within a defined academic unit.

For each included leader, the following variables were recorded: degree background, official leadership title, medical school type (public or private), institutional geographic region (Northeast, Midwest, South, West), and dean level (dean, vice dean, associate dean, assistant dean).

Degree background was coded into four mutually exclusive categories based on credentials listed in official biographies: MD/MBA holders (those with both MD and MBA degrees), MBA-only holders (those with an MBA but no MD), MD-only leaders (those holding an MD degree with no additional graduate degrees), and all others. Comparative analyses focused on the three prespecified groups of interest (MD/MBA, MBA-only, MD-only).

Leadership roles were classified according to their dominant functional portfolio. An inductive review of official titles and portfolio descriptions across institutions yielded 16 initial functional categories reflecting recurring administrative structures. Functional categories were derived based on similarities in official leadership titles and recurring portfolio language across institutions. For example, titles such as “Dean of Finance,” “Vice Dean for Finance and Administration,” and “Associate Dean for Fiscal Affairs” were grouped within a common finance-related category. Grouping decisions were based on the primary functional emphasis reflected in the official title and accompanying institutional description. Each leader was assigned to a single primary functional category based on dominant portfolio emphasis. Categories were designed to be mutually exclusive, and individuals were not assigned to more than one leadership domain to preserve statistical independence.

Although the 16 initial categories captured granular distinctions in administrative portfolios, many represented closely related functional areas (e.g. undergraduate medical education and graduate medical education) that would result in sparse cells within degree subgroups, particularly the MD/MBA group. Consolidation into six broader domains preserved conceptual similarity while improving statistical stability.

The 16 categories were therefore consolidated into the following six leadership domains prior to statistical analysis:

1. System Leadership, including institutional operations, finance, and executive administration portfolios;
2. Education Leadership, including student affairs/admissions, undergraduate medical education, graduate medical education, curriculum and assessment, and faculty affairs;
3. Clinical Affairs Leadership, including oversight of clinical services or faculty practice;
4. Research and Innovation Leadership, including research development or innovation portfolios;
5. Diversity, Equity, Inclusion, (DEI) and Community Engagement Leadership;
6. Special Projects and Other Administrative Roles, including cross-cutting or institution-specific initiatives.

The System Leadership domain included dean-level roles within the medical school with primary responsibility for finance, operations, strategy, or executive administrative oversight. This designation refers to school-level executive functions and does not include leadership roles within affiliated health systems. This grouping structure was finalized before inferential testing to preserve analytic integrity and avoid post hoc reclassification.

All extracted data were entered into a predefined spreadsheet template. Coding decisions were reviewed by both investigators, and discrepancies were resolved through discussion and consensus prior to statistical analysis.

Descriptive statistics were used to summarize the distribution of educational groups across leadership domains. To test for overall differences in leadership placement across degree groups, a 3 × 6 contingency table was constructed and analyzed using a Pearson chi-square test. Given small counts in some cells, pairwise comparisons were performed using a dichotomized outcome of system-facing versus non-system facing roles and analyzed with Fisher’s exact test. These pairwise comparisons were prespecified and limited to comparisons between the three degree groups; therefore, formal adjustment for multiple comparisons was not applied. System-facing roles corresponded to the System Leadership, whereas all other leadership domains were classified as non-system facing roles. Odds ratios and 95% confidence intervals were calculated from these 2 × 2 contingency tables. Statistical significance was defined as *p* < 0.05.

Exploratory analyses were conducted to compare institutional characteristics (public vs. private status, geographic region, and dean level) across degree groups. Chi-square tests were used where appropriate. All statistical analyses were performed using Microsoft Excel.

## Results

A total of 1,300 individuals were initially screened across the top 50 NIH-funded US medical schools. After applying exclusion criteria, 634 senior leaders met inclusion criteria and were included in the final cohort for further analysis. Of these, 22 were MD/MBA holders, 38 were MBA-only leaders, and 574 were MD-only leaders. Demographic characteristics did not differ significantly across degree groups (Table 1).

**Table 1:** Demographic characteristics of senior academic leaders by degree group

Characteristic	MD/MBA (*n* = 22)	MBA-only (*n* = 38)	MD-only (*n* = 574)	*p*-value*
School Type	10 Public / 12 Private	19 Public / 19 Private	312 Public / 262 Private	0.64
Region	5 Northeast / 3 Midwest / 8 South / 6 West	11 Northeast / 7 Midwest / 12 South / 8 West	150 Northeast / 132 Midwest / 172 South / 120 West	0.92
Leadership Level	1 Dean / 4 Vice / 9 Associate / 8 Assistant	3 Dean / 6 Vice / 14 Associate / 15 Assistant	50 Dean / 88 Vice / 210 Associate / 226 Assistant	0.99

* *p*-values were calculated using Pearson chi-square tests comparing MD/MBA, MBA-only, and MD-only leaders. No demographic variables differed significantly across groups

**Fig. 1:**
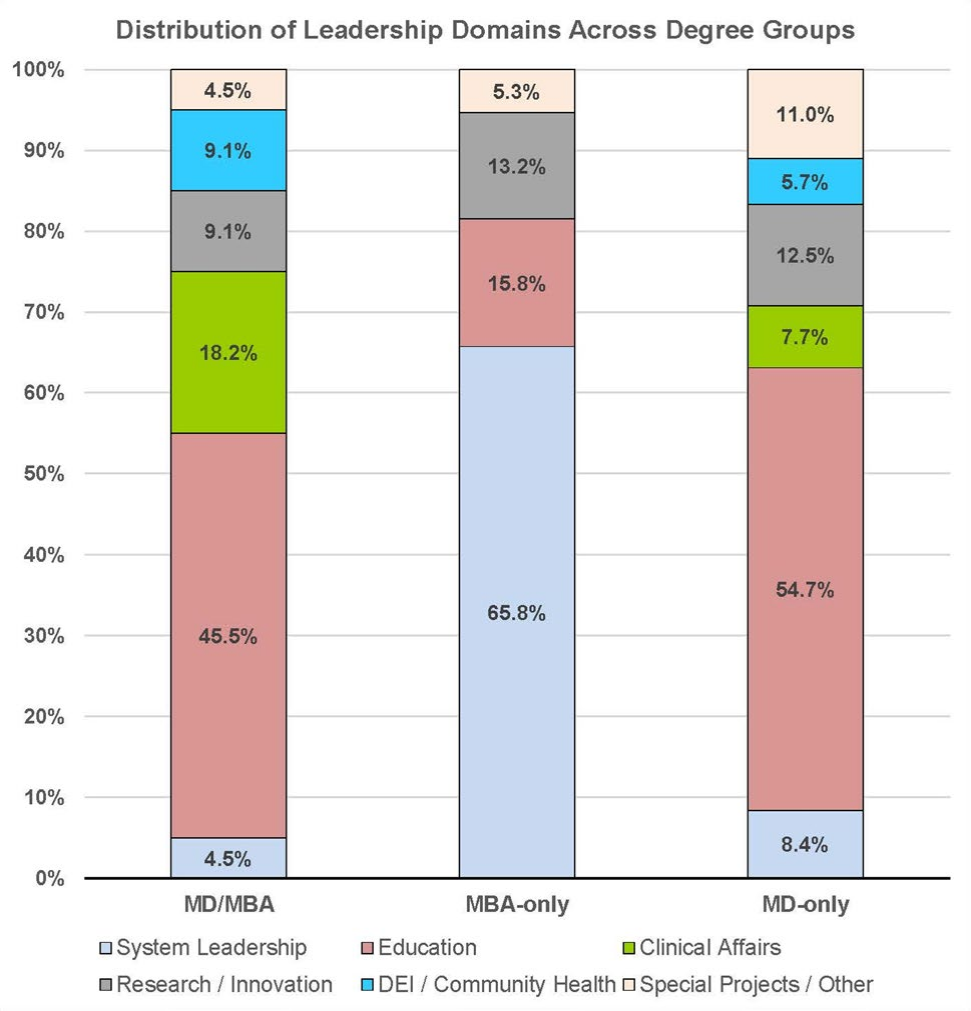
Proportion of leadership roles across six functional domains among MD/MBA (*n* = 22), MBA-only (*n* = 38), and MD-only (*n* = 574) leaders at the 50 highest NIH-funded US medical schools. Percentages represent the proportion within each educational group assigned to each leadership domain

Leadership role placement differed significantly across the three educational groups (χ²(10) = 119.5, *p* < 0.0001). Counts of leaders within each of their 16 original functional categories and their collapsed six-domain structure are presented in Table 2. As shown in Fig. 1, MD/MBA graduates most frequently held roles in educational and training-oriented domains, with nearly half (10/22, 45.5%) serving in roles related to “Education” and additional representation in “Clinical affairs” (4/22, 18.2%). Three MD/MBA leaders (3/22, 13.6%) held positions in “System leadership”. MBA-only administrators demonstrated a different distribution across leadership domains. 25 of 38 MBA-only leaders (65.8%) held system-facing roles involving institutional operations, financial oversight, or administrative strategy, with far fewer represented in educational or student-facing domains. MD-only leaders were distributed across multiple domains but were most heavily represented in the “Education” domain (314/574, 54.7%), followed by “Research and/or innovation” (72/574, 12.5%), with 48 MD-only leaders holding system-facing positions (48/574, 8.4%).

**Table 2:** Functional leadership categories and collapsed domains by degree group (raw counts underlying Fig. 1)

Original Category	Description	Domain	MD/MBA	MBA-only	MD-only
Operations, Finance, & Administration	Manages internal operations including human resources, budget, facilities, and institutional logistics	System Leadership	1	24	3
Executive Leadership	Holds institution-wide authority, often including Deans, Senior Vice Deans, or leaders of major school-wide strategy	System Leadership	2	1	45
Student Affairs & Admissions	Oversees student support services, admissions, and academic advising	Education	7	2	111
General Education	Oversees high-level medical education strategy including course planning, academic standards, and faculty-led instruction across preclinical and clinical domains	Education	0	2	51
Undergraduate Medical Education	Directs the four-year MD program including academic scheduling, course leadership, and day-to-day institutional operations	Education	0	1	44
Faculty Affiars	Oversees faculty recruitment, promotion, appointments, and career lifecycle processes	Education	0	1	42
Graduate Medical Education	Supervises residency and fellowship programs including accreditation, policies, and training quality	Education	3	0	40
Postdoctoral & Graduate Training	Oversees research-focused training programs for graduate students and postdoctoral fellows	Education	0	1	4
Cirriculum & Assessment	Develops and monitors the medical school curriculum, student learning objectives, and performance evaluations	Education	0	0	26
Clinical Affairs	Manages clinical operations, hospital affiliations, and patient care initiatives	Clinical Affairs	4	0	44
Research Leadership	Leads overarching research strategy, infrastructure, and institutional scientific direction	Research / Innovation	2	1	57
Innovation & Technology	Oversees digital transformation, tech-based initiatives, and innovation in medical education or care	Research / Innovation	0	3	11
Diversity, Equity, & Inclusion	Leads diversity, equity, inclusion and belonging efforts across faculty, staff, and students	DEI / Community Health	2	0	21
Global & Community Health	Coordinates partnerships, outreach, and programs in underserved communities locally and abroad	DEI / Community Health	0	0	12
Special Projects / Institutional Initiatives	Leads time-bound or strategic cross-functional initiatives designed by institutional leadership	Special Projects / Other	1	1	13
Other / Unclassified	Roles that do not fit into the primary categories listed or lack a clear functional designation	Special Projects / Other	0	1	50

To further evaluate leadership placement, pairwise comparisons were performed using a dichotomized outcome of system-facing versus non-system facing roles. The results of this analysis are displayed in Table 3. MD/MBA graduates did not differ significantly from MD-only leaders in their likelihood of holding a system-facing leadership position (odds ratio [OR] 1.73, 95% CI 0.49–6.06; *p* = 0.39). In contrast, MBA-only leaders were substantially more likely than MD/MBA graduates to hold system-facing roles (OR 12.18, 95% CI 3.03–48.9; *p* = 0.0004) and were also significantly more likely than MD-only leaders to do so (OR 21.07, 95% CI 10.12–43.85; *p* < 0.0001). Confidence intervals for comparisons involving the MD/MBA subgroup were wide, reflecting limited precision associated with small cell counts.

**Table 3:** Pairwise comparisons for system-facing roles

Comparison	OR*	95% CI	*p*-value
MD/MBA vs. MD-only	1.73	0.49–6.06	0.39
MBA-only vs. MD/MBA	12.18	3.03–48.9	0.0004
MBA-only vs. MD-only	21.07	10.12–43.85	< 0.0001

*Odds ratios reflect dichotomized comparison of System Leadership versus all other domains combined

Demographic characteristics did not differ significantly across the three degree groups. School type (χ²(2) = 0.90, *p* = 0.64), geographic region (χ²(6) = 2.04, *p* = 0.92), and leadership level (χ²(6) = 0.72, *p* = 0.99) were similar among MD/MBA, MBA-only, and MD-only leaders (Table 1). Chi-square assumptions were met for all demographic analyses, and therefore Fisher’s exact tests were not required. These findings suggest that demographic or institutional composition did not account for the substantial differences in leadership placement observed across groups.

## Discussion

This cross-sectional study examined the distribution of senior medical school leadership roles across degree backgrounds at the 50 highest NIH-funded U.S. medical schools. Leadership placement differed significantly across educational groups, with MBA-only leaders disproportionately represented in system-facing roles, whereas MD/MBA graduates were distributed more similarly to MD-only peers across educational, clinical, and academic domains.

Importantly, this study describes observed patterns of leadership placement and does not assess causality. Appointment to dean-level roles reflects a dynamic interplay between individual professional trajectories—including accumulated clinical, academic, and administrative experience—professional aspirations, and institutional selection processes. The present analysis does not evaluate these mechanisms but instead documents how leadership domain placement differs across degree backgrounds within this defined institutional context.

MD/MBA leaders (3/22, 13.6%) were not more likely than MD-only leaders (48/574, 8.4%) to occupy system-facing roles. In contrast, MBA-only leaders (25/38, 65.8%) were substantially more concentrated in system leadership domains. These findings describe the current distribution of dean-level appointments within research-intensive medical schools and should not be interpreted as direct measures of educational preparedness or managerial competence.

Prior scholarship has emphasized that background training and institutional context may influence pathways into senior academic leadership roles [13]. Similarly, frameworks in medical education highlight the role of professional identity formation and organizational culture in shaping leadership trajectories [7–9]. The present study does not directly test these theories but situates observed placement patterns within this broader conceptual landscape.

Survey-based studies have reported that physicians who pursue business education perceive their management training as relevant to administrative and leadership responsibilities [14]. However, leadership appointment reflects longitudinal professional experience and institutional criteria in addition to formal degree attainment. Accordingly, the present analysis examines the distribution of current leadership roles but does not assess institutional decision-making, individual career intent, or accumulated operational expertise. These descriptive findings may inform ongoing discussions among prospective trainees and program leaders regarding long-term leadership trajectories associated with different degree pathways.

The relatively small number of MD/MBA leaders (*n* = 22) warrants caution in interpreting subgroup comparisons. Sparse cell counts contributed to wide confidence intervals for certain odds ratio estimates, limiting precision. Although significant differences in system-facing role placement were observed, effect size estimates involving the MD/MBA subgroup should be interpreted descriptively and with appropriate caution.

Several additional limitations merit consideration. First, the cross-sectional design precludes assessment of career trajectories, temporal trends, or causal relationships. Second, data were derived from publicly available institutional websites and biographies, which may incompletely reflect functional authority or recent leadership transitions. Leadership titles may not fully reflect functional authority or scope of responsibility, introducing potential misclassification bias. Third, the analysis was intentionally restricted to senior leaders within medical schools at the 50 highest NIH-funded institutions and does not evaluate leadership roles within affiliated health systems, faculty practice plans, community hospitals, or international settings. These findings therefore may not generalize beyond similarly structured research-intensive medical schools.

Finally, MD/MBA programs remain relatively recent compared with the extended professional timelines typically required to attain dean-level appointments. The current cross-sectional snapshot may therefore not reflect the eventual representation of dual-degree graduates in senior administrative positions. Leadership composition at medical schools may evolve gradually, and longitudinal follow-up would be required to determine whether patterns of degree representation change over time as additional dual-degree cohorts mature professionally.

Future research using longitudinal designs or mixed-methods approaches may help clarify how degree attainment interacts with professional experience, institutional culture, and leadership selection processes. Such work may provide a more comprehensive understanding of how dual-degree pathways are reflected in long-term leadership outcomes within academic medicine.

## Conclusions

In this cross-sectional analysis of senior leadership within the 50 highest NIH-funded US medical schools, leadership role placement differed significantly across degree backgrounds. MBA-only leaders were disproportionately represented in system-facing roles, whereas MD/MBA graduates were distributed more similarly to MD-only peers across educational, clinical, and academic domains.

These findings describe current patterns of leadership role distribution within research-intensive US medical schools and do not assess leadership effectiveness, institutional decision-making processes, or individual career intentions. Leadership placement reflects the interaction of accumulated professional experience, organizational context, and institutional selection, factors not directly measured in this analysis.

Future longitudinal designs following dual-degree cohorts over time, as well as mixed-methods studies examining institutional decision-making and individual career trajectories, will be necessary to more fully understand how degree attainment and accumulated experience interact in shaping senior leadership pathways within academic medicine.

## Data Availability

The data that support the findings of this study are available from the corresponding authors upon reasonable request.

## Abbreviations

DEI: Diversity, equity, and inclusion
MBA: Master of Business Administration
MD: Doctor of Medicine
NIH: National Institutes of Health
US: United States
